# SAPAP Scaffold Proteins: From Synaptic Function to Neuropsychiatric Disorders

**DOI:** 10.3390/cells11233815

**Published:** 2022-11-28

**Authors:** Yunxia Bai, Huimin Wang, Chunxia Li

**Affiliations:** 1 Key Laboratory of Brain Functional Genomics (STCSM & MOE), Affiliated Mental Health Center (ECNU), School of Psychology and Cognitive Science, East China Normal University, Shanghai 200062, China; 2 Shanghai Changning Mental Health Center, Shanghai 200335, China; 3 NYU-ECNU Institute of Brain and Cognitive Science at NYU Shanghai, Shanghai 200062, China

**Keywords:** SAPAP/DLGAP/GKAP, postsynaptic scaffolding protein, excitatory synapse, animal model, cognitive dysfunction, neuropsychiatric disorders

## Abstract

Excitatory (glutamatergic) synaptic transmission underlies many aspects of brain activity and the genesis of normal human behavior. The postsynaptic scaffolding proteins SAP90/PSD-95-associated proteins (SAPAPs), which are abundant components of the postsynaptic density (PSD) at excitatory synapses, play critical roles in synaptic structure, formation, development, plasticity, and signaling. The convergence of human genetic data with recent in vitro and in vivo animal model data indicates that mutations in the genes encoding SAPAP1–4 are associated with neurological and psychiatric disorders, and that dysfunction of SAPAP scaffolding proteins may contribute to the pathogenesis of various neuropsychiatric disorders, such as schizophrenia, autism spectrum disorders, obsessive compulsive disorders, Alzheimer’s disease, and bipolar disorder. Here, we review recent major genetic, epigenetic, molecular, behavioral, electrophysiological, and circuitry studies that have advanced our knowledge by clarifying the roles of SAPAP proteins at the synapses, providing new insights into the mechanistic links to neurodevelopmental and neuropsychiatric disorders.

## 1. Introduction

Synapses are fundamental elements of neural circuits and networks that convey all aspects of brain function, and pathological alterations in synaptic structure and function are broadly held to underlie many neuropsychiatric disorders, such as autism spectrum disorder (ASD), schizophrenia, obsessive compulsive disorder (OCD), cognitive disorders, and mood disorders [1,2,3,4,5,6,7]. In the mammalian brain, the vast majority of synapses are excitatory synapses (primarily glutamatergic), which occur predominantly at contacts between presynaptic axons and postsynaptic tiny, actin-rich protrusions known as dendritic spines. During synaptic plasticity at excitatory synapses, the molecular composition of postsynaptic membranes and the chemical modification of synaptic proteins are key determinants of the number, strength, morphology, and even connectivity of neuronal synapses [8,9,10]. The postsynaptic density (PSD) at the tip of a dendritic spine’s head, which is an electron-dense thickening assembly situated underneath the postsynaptic membrane, is composed of numerous proteins, including adhesion proteins, membrane-tethered receptor and ion channels, scaffold proteins, signaling molecules, and cytoskeletal proteins [8,9,11]. These components of the PSD assemble into a dynamic macromolecular complex and are crucial for synaptic transmission and plasticity [12]. During development, and in response to stimulation or inhibition, the PSD dynamically undergoes changes in molecular composition and structure in order to tune the strengths and/or efficacy of synaptic signaling.

**Figure 1:**
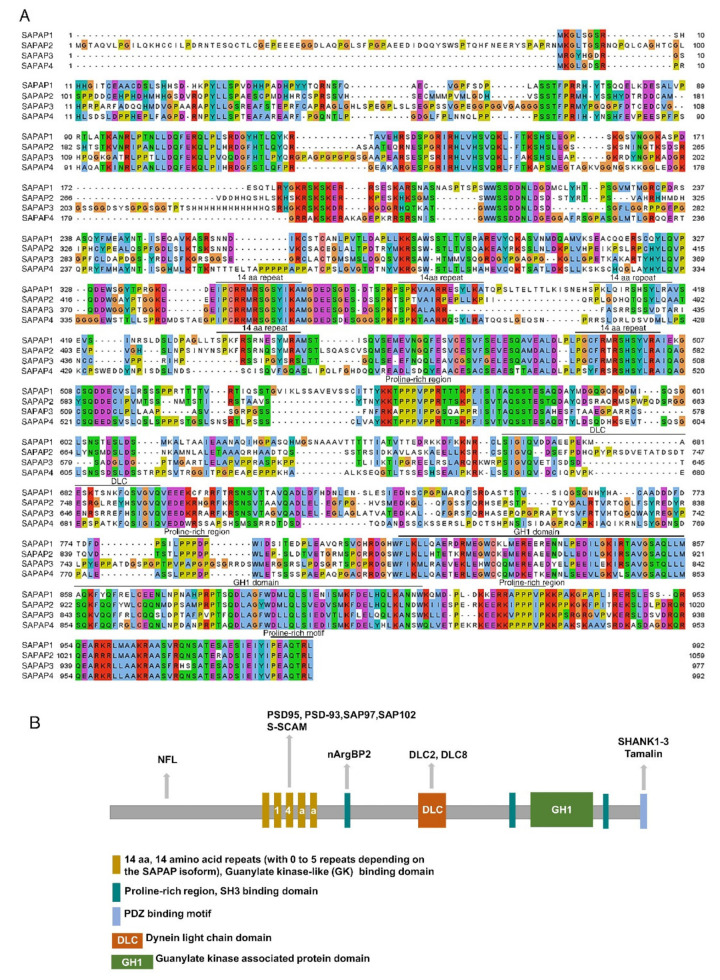
Structure and domains of SAPAP proteins: (**A**) Amino acid sequence alignment of SAPAPs—SAPAP1 isoform a: 992 amino acids (NP_808307.2); SAPAP2 isoform a: 1059 amino acids (NP_766498.2); SAPAP3: 977 amino acids (NP_001289010.1); SAPAP4 isoform a: 992 amino acids (NP_666240.4). The alignment was generated and visualized using Jalview 2.11.2.0. The potential interacting domains are marked with black lines. (**B**) Only a subset of known binding partners is shown.

The SAPAP (SAP90/PSD-95-associated protein, also called discs-large-associated proteins (DLGAPs)) family is made up of key postsynaptic scaffold proteins that are highly concentrated in the PSD of excitatory synapses and are essential for synaptic structure and functions [1,4,8,13,14,15,16,17]. In the brain, the SAPAP family is composed of four proteins encoded by four homologous genes (Figure 1A), which have different alternative splicing variants [4]: *DLGAP1* (also referred to as guanylate kinase-associated protein *GKAP* and *SAPAP1*, located on chromosome 18p11), *DLGAP2* (also known as *SAPAP2*, 8p23), *DLGAP3* (also known as *SAPAP3*, 1p34), and *DLGAP4* (also known as *SAPAP4*, 20q11). Owing to their differential expression in the brain and simultaneous expression in many brain regions [18,19], the different members of the SAPAP family not only play distinct physiological roles, but also cooperate to play physiological roles in excitatory synapses.

Several groups have reported changes in the expression and epigenetic dysregulation of *SAPAP* genes in patients afflicted with schizophrenia, alcohol use and dependence, Alzheimer’s disease (AD), or cerebellar ataxia [20,21,22,23]. Recent studies have also found that copy number variants (CNVs) and single-nucleotide polymorphisms (SNPs) in diverse neuropsychiatric disorders such as ASD, OCD, and schizophrenia are present in the *SAPAP* family genes [2,22,23,24,25,26,27,28,29,30,31,32,33,34]. Further studies with *Sapap1–4*-mutant mice have revealed that these mice show marked behavioral abnormalities relevant to neuropsychiatric disorders, including increased repetitive behavior and anxiety, hyperactivity, mania-like phenotypes, altered memory ability, decreased alcohol use, and altered sensitivity to psychostimulants [13,14,15,16,17,21,35].

Despite the potential importance of SAPAP proteins in these neuropsychiatric disorders, the in vivo functional roles of SAPAP proteins are not yet fully understood. Here, we review the most recent findings that have emerged from human genetics studies exploring the links between mutations in *SAPAP* genes and neuropsychiatric disorders. We further review evidence from the recent studies of mutant mice concerning the physiological roles of SAPAP proteins at the excitatory synapses and provide deeper insights into the mechanistic links to neuropsychiatric disorders.

## 2. SAPAP Family

The SAPAP family proteins were originally identified as synaptic proteins that interact with the PSD-95 family and termed SAP90/PSD95-associated proteins [36,37]. The full-length structure of SAPAP proteins is characterized by a 14-amino-acid (aa) repeat domain, a dynein light-chain (DLC) domain, three proline-rich domains, and a GKAP homology (GH1) domain [36,37,38], by which SAPAPs bind to their interacting partners (Figure 1B).

### 2.1. SAPAP-Interacting Proteins

With the N-terminal conserved 14 aa repeat domain, SAPAP proteins directly interact with the GK domain of the PSD-95 family scaffolding proteins—a subfamily of the membrane-associated guanylate kinase (MAGUK) family composed of PSD-95, PSD-93, SAP97, and SAP102 [1,36]. PSD-95 and other members of the MAGUK family not only interact directly with the NR2A and NR2B subunits of NMDARs and Shaker-type K^+^ channels through C-terminal PDZ ligand motifs to promote the clustering of these proteins in the PSD [1,11], the neuronal cell adhesion molecule neuroligin [39,40] via its third PDZ domain, but also interact indirectly with AMPA receptors (AMPARs) through interaction with the transmembrane protein stargazin/TARPs [41,42,43], or other auxiliary proteins [44] to regulate the synaptic trafficking of NMDARs and AMPARs [45,46]. Signaling molecules including SynGAP (a synaptic Ras-GTPase activating protein) and neuronal nitric oxide synthase (nNOS) also interact with the PDZ domains of PSD-95 and present in a large complex with PSD-95 and the NMDARs in the brain [47,48]. Both the SH3 and GK domains in PSD-95 bind to and induce clustering of the kainate subtype ionotropic glutamate receptors [49], which have been linked to schizophrenia and other psychiatric disorders, such as intellectual disability (ID) and ASD [50].

An additional partner to the 14 aa repeat domain of SAPAP proteins is S-SCAM [51], which also interacts with NMDAR subunits, neuroligin 1, and β-catenin at excitatory synapses [51,52], neuroligin 2 and β-dystroglycan at inhibitory synapses [52], and the scaffold protein tamalin [53]. In addition, the N-terminal region of SAPAPs also interacts with neurofilaments (NFLs), but not with actin or tubulin. The NFL-interacting region is different from the regions interacting with PSD-95 and S-SCAM [54].

The C-terminal region of SAPAP proteins subsequently interacts with the PDZ domain of the SHANK protein family, including SHANK1, SHANK2, and SHANK3 [55,56]. SHANKs then not only bind to the Homer family proteins—thereby linking metabotropic glutamate receptors (mGluR1/5) to SAPAPs [57]—but also provide a link between the SAPAP proteins and the actin cytoskeleton through interactions with the F-actin-binding protein cortactin [55,58]. SAPAPs can bridge the PSD-95 family and SHANK family to form the PSD-95/SAPAPs/SHANKs core complex [36], which is thought to be a major scaffold organizer in orchestrating the synaptic formation and plasticity at glutamatergic synapses. Additionally, SAPAP–SHANK interaction also involves the participation of N-terminal extension sequences of the PDZ domain [59,60]. These extension sequences govern the exquisite and strong interaction between SAPAPs and SHANKs. A recent study also shows that PSD-95, SAPAP1, and SHANK3 can form scaffold complexes with one another at different developmental stages [61].

The proline-rich domain of SAPAPs binds to the third SH3 domain of nArgBP2 [62]. nArgBP2, which is important for neuronal dendritic development and spine synapse formation [63,64], then binds to signaling molecules including the ubiquitin ligase CBL, protein kinases ABL and PYK2, and various proteins involved in the regulation of cell adhesion and the actin cytoskeleton [63,64,65].

The DLC domain of SAPAP proteins binds directly to DLC2 and DLC8 [38,66,67], which colocalize with SAPAP/PSD-95 in the PSD and also tend to distribute into the deep part of the spine [38]. DLC is an accessory subunit shared by dynein and myosin-V, and is highly enriched in dendritic spines [38,67], suggesting that DLC may link SAPAPs to actin- and microtubule-based motors and act as a motor–cargo adaptor.

The structure of the GH1 domain of SAPAP proteins is believed to be dynamic [68]. The function and interacting partners of the GH1 domain have not yet been well described [36]; however, one recent study identified a patient with cortical malformations who carried a novel *SAPAP4* mutation, affecting the last part of the GH1 domain and leading to loss of a polyproline-rich domain of SAPAP proteins [34].

In addition to the aforementioned interacting partners, a number of other proteins have been identified as SAPAP-interacting proteins, including the scaffold protein tamalin (binding to the C-terminal PDZ-binding motif of SAPAP1/3) [53], nNOS (identified as a SAPAP1-interacting protein in HEK 293 human kidney epithelial cells [67], echinoderm microtubule-associated protein-like 1 (EML1), LGN, cortactin (actin cytoskeleton-regulating protein), β-catenin (SAPAP4-interacting protein, identified in Neuro2A cells) [34], spinophilin (SAPAP3 interacting protein) [69], and cyclin-dependent kinase-5 (CDK5) [70].

### 2.2. Expression of SAPAPs in the CNS

During postnatal development, the different SAPAP members are expressed differently. The temporal patterns of *Sapap1* and *Sapap2* expression in individual brain regions do not change prominently after birth [19]. For example, the expression of *Sapap1* mRNAs in the cortex seems to slightly peak around postnatal day 90. However, the spatiotemporal expression pattern of *Sapap3* during early postnatal development is noticeable, with peak expression observed around 2–3 weeks after birth in the cortex, striatum, and thalamus [19,71]. Although high expression levels are seen in the cortex within the first 3 weeks after birth, the distribution of *Sapap4* mRNA appears to slightly decline afterwards [19]. Additionally, recent studies have indicated that both *Sapap1* and *Sapap4* are expressed in progenitor cells—the latter notably strongly expressed in the cortical progenitors and migrating neurons—suggesting the important roles of SAPAPs during early corticogenesis [34,72].

In the adult rodent brain, the *Sapap* mRNAs and proteins display overlapping yet distinct distribution characteristics. All four *Sapap* mRNAs are expressed abundantly in the cerebral cortex, hippocampus, and olfactory bulb, but at low or undetectable levels in the hypothalamus and substantia nigra [18,19]. *Sapap1* mRNA is expressed abundantly in the cortex, hippocampus, amygdala, cerebellum, and olfactory bulb, moderately in the thalamus, and at low levels in the striatum [13,18]. Notably, *Sapap1* mRNA is only expressed in a small subset of cells in the striatum and displays heterogeneous expression patterns in different subregions of the thalamus [18], but it is uniformly distributed throughout the whole cerebral cortex.

Distinct from the other SAPAP family members, *Sapap2* mRNAs are expressed in a limited pattern—mainly in the forebrain, with relatively low expression levels. *Sapap2* displays its highest expression levels in the hippocampus, with moderate levels in the striatum and almost undetectable levels in the cerebellum, thalamus, and amygdala [13,18]. In the cortex, *Sapap2* mRNAs show relative strong expression in layers 2 and 3 [18].

The most abundant *Sapap* detected in the striatum and various thalamic nuclei is *Sapap3* [18,19]. Moreover, *Sapap3* mRNAs are also highly expressed in layers 1–3 of the cortex, but only moderately expressed in the amygdala [13,18]. An intriguing aspect of the subcellular distribution of *Sapap3* is its dendritic localization. It has been found that several mRNAs are localized in the dendrites [73,74], and an extensive body of literature directly linked synaptic plasticity with local protein synthesis and degradation in dendrites [75,76,77] (reviewed in [73,78,79,80]). In the hippocampus, *Sapap3* is the only family member that is highly localized in dendrites [18,19], which possibly allows rapid turnover of proteins and thus provides a mechanism for quick, activity-dependent changes in synaptic protein complex composition [73,80,81], suggesting that SAPAP3 may play a unique role in synaptic plasticity.

*Sapap4* is highly expressed in many regions of the brain, such as the hippocampus, cortex, amygdala, and cerebellum [13,18,19]. In the hippocampus, the expression of *Sapap4* mRNA and proteins is relatively high in CA1 and CA3, but low in the dentate gyrus. Additionally, *Sapap4* mRNA shows high expression in layer 5 of the cortex. In addition to these areas, *Sapap4* is also strongly expressed in the adult rodent thalamus [17,18] and striatum [13,82]—second only to *Sapap3*. Of particular interest, *Sapap4* mRNA is strongly expressed in the locus coeruleus [18] as well as the ventricular zone and migrating neurons [34].

In general, the SAPAP proteins are predominantly localized at glutamatergic and cholinergic synapses, and not at GABAergic or glycinergic synapses [18], suggesting that SAPAPs may be a general “core” postsynaptic component of excitatory synapses, but not of inhibitory synapses. Moreover, subsequent studies have found that SAPAP3 and SAPAP4 may tend to present at corticostriatal and thalamostriatal excitatory synapses, respectively [13,82]. These findings of molecular heterogeneity of SAPAPs at different synapses imply that SAPAP family members may be localized at synapses in a circuit-selective manner, and that the molecular specificity in SAPAP proteins attributes a unique and specific function to the SAPAP family members. A systematic analysis of the SAPAPs according to the subregions and developmental stages of the brain remains to be determined and would be important for understanding the functions of SAPAPs in the brain.

## 3. Roles of SAPAPs in Synaptic Structure and Function

**Figure 2:**
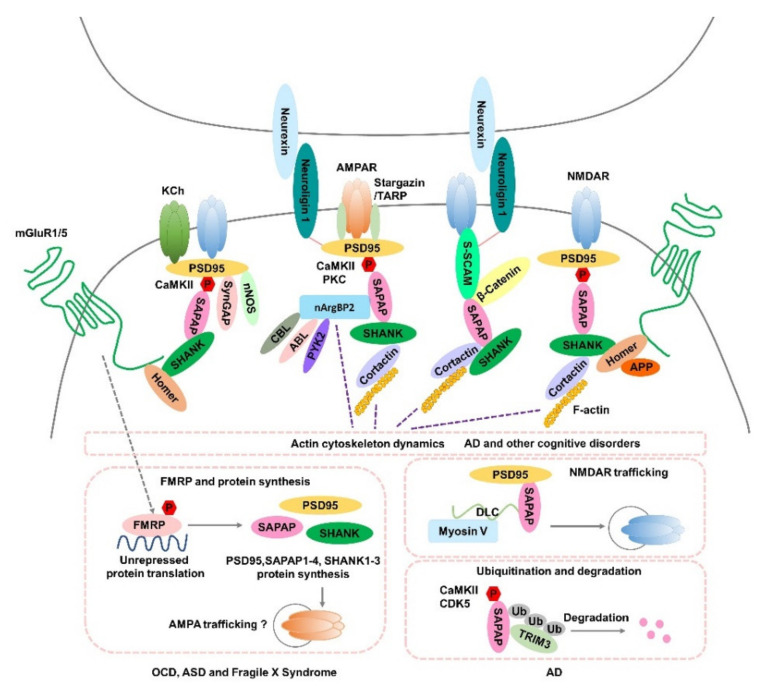
Potential roles of SAPAPs in synaptic function and the pathogenesis of neuropsychiatric disorders. Hypothesized mechanisms of SAPAPs for the molecular signaling underlying OCD, ASD, Fragile X Syndrome, AD and other cognitive disorders are summarized in the figure. AMPAR, α-amino-3-hydroxy-5-methyl-4-isoxazole propionic acid receptor; NMDAR, N-methyl-D-aspartic acid receptor; KCh, Shaker-type K^+^ channels; SAPAP, SAP90/PSD-95-associated protein; mGluR1/5, Group I metabotropic glutamate receptors; CaMKII, Calcium/calmodulin-dependent protein kinase II; PKC, Protein kinase C; SynGAP, Synaptic GTPase Activating Protein; nNOS, Neural Nitric Oxide Synthase; S-SCAM, synaptic scaffolding molecule; nArgBP2, Neural Abelson-related gene-binding protein 2; CBL, E3 ubiquitin ligase casitas B-lineage lymphoma; ABL, tyrosine kinase; PYK2, proline-rich tyrosine kinase 2; APP, amyloid precursor protein; P, phosphorylation; FMRP, fragile X mental retardation protein; TRIM3, Tripartite motif-containing 3; Ub, ubiquitin; DLC, dynein light-chain; CDK5, cyclin-dependent kinase 5; TRAP, transmembrane AMPAR regulatory protein.

SAPAPs play direct or indirect roles in cellular signaling. Through their interactions with PSD-95 and SHANKs/Homers, SAPAPs couple the neuronal cell adhesion molecule neuroligin [39,40], ionotropic glutamate receptors, and ion channels—including NMDARs [59], AMPARs [41,42], kainate-type glutamate receptors [49], and Shaker-type K^+^ channels [83]—with metabotropic glutamate receptors (mGluR1/5) and other downstream signaling proteins, bridging the PSD and the actin cytoskeleton and linking multiple cellular pathways together [9,11,84]. Conversely, SAPAPs can also directly recruit signaling molecules such as nNOS and nArgBP2—a modulator of both cell adhesion and the actin cytoskeleton [63,64,65]. Interestingly, the overexpression of *Sapap4* in human retina pigment epithelial cells also disturbs neuronal migration and impacts actin cytoskeleton dynamics, probably involving cortactin and F-actin [34] (Figure 2).

The SAPAPs themselves also act as substrates for phosphorylation and ubiquitination. Previous in vivo and in vitro studies have indicated that SAPAPs can be phosphorylated by kinases such as PKA, PKC, CDK5, ERK1, P38 MAPK, AKT1, and CaMKII [70,84,85,86]. SAPAP1 protein complexes show increased binding in specific kinases and phosphatases upon induction of LTP [87]. Interestingly, PSD-95–SAPAP interaction and SAPAP–DLC interaction, which are dependent upon CaMKII phosphorylation of SAPAP, are crucial for proper SAPAP targeting and synaptogenesis [86,88]. Moreover, PKC phosphorylation of SAPAP proteins can also promote PSD-95–SAPAP complex formation and further enhance the clustering and formation of AMPAR nanodomains [84], while CaMKII phosphorylation of SAPAPs can control the turnover of SAPAPs at synapses in a bidirectional manner and phosphorylation of Ser54 in SAPAP promotes its removal from synapse sites [86]. Notably, overexpression of the SAPAP turnover mutants not only results in the loss of polyubiquitination and degradation of SAPAP from synapses but also eliminates activity-dependent remodeling of PSD-95, SHANK and regulation of surface AMPAR levels [86], indicating that SAPAP is critical for regulation of postsynaptic protein organization and synaptic structure. Moreover, SAPAPs can also be ubiquitinated by an E3 ligase (TRIM3), leading to its subsequent degradation and loss of SHANK1 from postsynaptic sites, which is involved in regulation of dendritic spine morphology [89]. CDK5 phosphorylation of SAPAP appears to be central in triggering ubiquitination and degradation of SAPAPs, as well as remodeling of the synaptic actin structures [70] (Figure 2). Together, the specialized functional network organized by PSD-95–SAPAP–SHANK scaffold complexes facilitates the activity-dependent remodeling of dendritic spines [86,89]; thus, the disruption of the activity-dependent turnover of PSD scaffold proteins such as SAPAPs can affect synaptic formation and plasticity, leading to abnormal synaptic and behavioral phenotypes.

### 3.1. Synaptic Formation and Maturation

As an indispensable central member of the axis of PSD-95–SAPAP–SHANK scaffold complexes in the PSD [4,8,9,11], SAPAPs play critical roles in initial synaptic formation and development. SAPAPs are always present before NMDAR and AMPAR clusters become synaptic [90], suggesting a structural role of SAPAPs in synapse formation. Indeed, the SAPAP–SHANK interaction is known to be essential for synaptic targeting of SHANK1 [55,91], whereas the interaction between SAPAPs and PSD-95 is crucial for synaptic localization of NMDARs [90], SHANKs [92], and SAPAPs [88]. Moreover, the SAPAP–DLC2 interaction is primarily located in dendritic spines, activity-dependent [93], and also thought to play important roles in proper targeting of SAPAP proteins to synapses [86] and controlling NMDARs’ function in synaptic transmission [94] (Figure 2).

Consistent with an essential function in SAPAP-mediated synaptogenesis and synaptic activity [88], the studies of SAPAPs in vivo have also highlighted a potential role of SAPAPs in synaptic development. For example, the deletion of the *Sapap3* gene results in an increase in “juvenile” NMDAR subunit composition [13] and silent synapses (i.e., those containing no AMPARs) [95], suggestive of immature corticostriatal synapses. Intriguingly, the absence of *Sapap4* also leads to similar alterations in the hippocampus [16], suggesting critical roles of SAPAP3 and SAPAP4 in promoting the early postnatal switch in NMDAR subunit expression and regulating the maturation of excitatory synapses.

Correspondingly, loss of SAPAPs in mice not only leads to disruption of protein interactions in the PSD [15], but also affects the dendritic arborization, spine numbers, axon caliber, and the structure of excitatory synapses in different brain regions [13,14,16,17,35,96]. In particular, SAPAP2 has been identified as a critical component involved in modulating synaptic function and development. For *Sapap4*, cell-type-specific differential isoform expression is seen during the transition from immature to mature neurons, probably reflecting the dynamic, diverse, and complex functions of SAPAPs [97]. More recently, SAPAP4 has been found to be required for proper cortical development [34]. These findings indicate an important role of SAPAPs in regulating excitatory synaptic formation and maturation.

### 3.2. Synaptic Transmission and Plasticity

Homeostatic plasticity has essential consequences for maintaining the stable function of neural circuits over a wide range of spatiotemporal scales [98]. There is evidence that SAPAPs can contribute to synaptic scaling by regulating the accumulation of postsynaptic receptors and scaffold proteins at synaptic sites [4,86]. The ubiquitination and degradation of SAPAPs at synapses is thought to be required for the normal activity-dependent remodeling of postsynaptic scaffold proteins such as PSD-95 and SHANKs, as well as excitatory synaptic scaling [86]. Consistent with the role of SAPAPs in synaptic scaling, deletion or perturbation of different SAPAP family members in mice also leads to alterations in the PSD levels of receptors and scaffold proteins, including NMDARs, AMPARs, mGluRs, Homer, SHANK, and αCaMKII [13,14,15,16,17,99]. Strikingly, not only the deletion of the repeat region R1 in SAPAP [86], but also the removal of SHANK [100], leads to the loss of AMPAR-containing synapses, along with impaired synaptic transmission.

The absence of SAPAP2 can lead to postsynaptic and presynaptic deficits, including a decrease in AMPAR-mediated postsynaptic response and an increased paired-pulse ratio (PPR), which indicates a reduction in the probability of presynaptic release in the orbitofrontal cortex (OFC) [14]. Two recent independent studies using different knockout strategies revealed that *Sapap4*-mutant mice exhibit decreased NR2A-mediated NMDAR currents and increased AMPAR transmission in the hippocampus [16], impaired synaptic transmission, and decreased AMPAR-mediated postsynaptic responses in the nucleus accumbens (NAc) [35], without alterations in the presynaptic neurotransmitter release. Moreover, SAPAP4 deficiency also impairs neuronal network function and affects synaptic plasticity in the hippocampal CA1 region, impairing long-term depression (LTD) but enabling the induction of long-term potentiation (LTP) [16].

Due to its strong association with OCD, the synaptic roles of SAPAP3 have been well studied. *Sapap3*-mutant mice exhibit significant deficits in corticostriatal synaptic function, including reduced AMPAR-mediated postsynaptic responses and elevated signaling through NMDAR and mGluR5 [13,95,99,101], without significantly affecting thalamostriatal AMPAR synaptic function [82]. Furthermore, there is ample evidence for input- or circuit-specific impairments of corticostriatal synaptic transmission [82,101,102,103,104,105,106,107]. Beyond participating in glutamatergic system, SAPAPs have been implicated in modulating the monoaminergic system. *Sapap3*-mutant mice display upregulation of serotonin turnover in cortical and striatal regions and dihydroxyphenylacetic acid/dopamine ratios in the OFC [108], along with alterations in dopamine receptor density within the NAc [109], indicating an important role of SAPAP proteins in glutamate–monoamine interplay, which is relevant in psychiatric disorders such as schizophrenia, mood disorders, and OCD [110,111]. A follow-up study revealed an anomalous excessive form of synaptic plasticity (i.e., endocannabinoid-mediated LTD) expressed at striatal excitatory synapses, requiring mGluR5 signaling in *Sapap3*-mutant mice [99].

## 4. SAPAPs’ Expression, FMRP, and Neuropsychiatric Disorders

Substantial studies have shown the genetic and epigenetic dysregulation of *SAPAP* genes in patients afflicted with psychiatric disorders or related animal models [21,22,23,112,113,114,115,116]. Fragile X mental retardation protein (FMRP) is considered to be a translational repressor that plays a key role in regulating local mRNA translation in response to synaptic signaling. In *Fmr1*-knockout (KO) mice—a model for fragile X syndrome—the protein levels of several FMRP targets are increased in PSD fractions either from the neocortex or hippocampus, including *Sapap1–3*, *Shank1*, *Shank3*, and various glutamate receptor subunits [117]. Conversely, *Sapap3* is decreased in both the OFC and medial prefrontal cortex (mPFC) of *Fmr1*-KO mice [118], which may contribute to the deficits in cognitive flexibility found in fragile X syndrome. Additionally, aggressive experience also increases the expression of *Sapap3*, coupled with a decrease in FMRP phosphorylation that is dependent on the activation of mGluR5 [119]. The postsynaptic targets of FMRP also include *Sapap4*, mGluR5, and many other scaffold proteins that comprise the glutamate receptor interactomes, such as PSD-95, Homer1, and *Neuroligins* 1–3 [120,121,122]. The mRNA for *Sapap4* is significantly increased by the activation of mGluRs in WT but not *Fmr1*-KO neurons [123]. Diminished mGluR-induced dendritic localization of *Sapap4* mRNA is found in the hippocampal neurons of *Fmr1*-KO mice [123], indicating a surprising coordinate regulation between *Sapap4* and mGluRs that may be altered in fragile X syndrome. In accordance with these findings, increased mGluR5-signaling-dependent AMPAR endocytosis or altered mGluR5–Homer scaffolds are observed in OCD models with *Sapap3*-mutant mice, fragile X syndrome models with *Fmr1*-KO mice, and autism models with *Shank3*-mutant mice, which all include repetitive behaviors, among other symptoms [95,101,124,125], suggesting a potential shared genetic mechanism of different neuropsychiatric disorders (Figure 2).

Altered expression levels of *SAPAPs* are also observed in different neuropsychiatric disorders (Table 1). *Sapap1* expression in the NAc is significantly increased in both animal models of phencyclidine-induced schizophrenia and unmedicated patients [115]. *Sapap2* mRNA expression, which is significantly associated with its DNA methylation status, was associated with coping with stress in an animal model of post-traumatic stress disorder (PSTD) [112]. Similarly, the effects of *Sapap2* methylation on memory ability in AD patients are also thought to be mediated by changes in *SAPAP2* expression in the dorsal lateral prefrontal cortex (dlPFC) [22]. Increases in *Sapap3* levels have been observed in both epilepsy patients and murine models [116]. *Sapap4* has been indicated to be involved in psychostimulants’ actions and bipolar disorder (BD). In taste-aversion-resistant rats after cocaine exposure, the mRNA levels of *Sapap4* in the amygdala were strikingly lower than those in taste-aversion-prone rats [126]. Remarkably, sensitivity to cocaine and amphetamine was also changed in *Sapap4*-deficient mice [17,35]. Interestingly, in human neural progenitor cells derived from BD patients, *Sapap4* has been identified as a target gene of miR-1908-5p-a BD-associated epigenetic regulator [127]—suggesting that epigenetic dysregulation of *SAPAP4* may be involved in the pathogenesis of BD.

Epigenetic dysregulation of the expression of *SAPAP* genes, mainly mediated by changes in DNA methylation and histone acetylation, may confer risks of various neuropsychiatric disorders (Table 2). Epigenetic modifications of the *Sapap2* gene have been implicated in PTSD [112], alcohol and cannabis use [21,114], schizophrenia [113], and AD [22]. One study revealed that histone deacetylases and methyl-CpG-binding protein 2 (*MeCP2*) inhibit repetitive behaviors through regulation of the *Sapap3* gene [128]. Additionally, recent studies have also reported an association between altered methylation of *SAPAP1* and stress responses to violence [129] and a link between epigenetic dysregulation of *SAPAP4* and early-onset cerebellar ataxia [23,130].

## 5. Human Molecular Genetic Analysis of Neuropsychiatric Disorders Linked to SAPAP

### 5.1. SAPAP1 and Neuropsychiatric Disorders

Several human molecular genetic studies have emerged to show that *SAPAP1* is related to several neuropsychiatric disorders, including schizophrenia, OCD, AD, ADHD, ASD, mood disorders, and cortical malformation [24,25,26,27,28,131,132,133,134] (Table 3). The *SAPAP1* gene is located on chromosome 18p11, which has been suggested to be a susceptibility region for schizophrenia and/or BD [135,136]. Although several SNPs found in *SAPAP1* are not associated with schizophrenia [131,132], a de novo CNV mutation of *SAPAP1* has been identified in patients with schizophrenia [24]. The first genome-wide association study (GWAS) with 1465 OCD cases revealed that the two SNPs with the lowest *p*-values were located within *SAPAP1*, suggesting a subthreshold statistical significance for *SAPAP1* [25]. Furthermore, the fact that two rare CNVs (a 62 kb duplication for *SAPAP1* and a 16 kb deletion for *SAPAP2*) overlapping the FMRP targets *SAPAP1* and *SAPAP2* were uncovered in another OCD study is of particular interest [26]. Two *SAPAP1* SNPs associated with cognitive flexibility have also been identified in ADHD children [27]. In addition, *SAPAP1* is also implicated in ASD [133] and recurrent major depressive disorder (MDD) [134]. Interestingly, the expression of *SAPAP1*, which is significantly downregulated in the hippocampus and cortex of AD patients, is strongly associated with early identified AD variants (i.e., noncoding SNP rs8093731 in *Desmoglein-2*/*DSG2* that acts as an expression quantitative trait loci for *SAPAP1*) [28] and *SAPAP1* is identified as a potential candidate gene involved in AD.

### 5.2. SAPAP2 and Neuropsychiatric Disorders

Numerous studies have implicated *SAPAP2* as being the strongest candidate gene for neurodevelopmental or behavioral phenotypes and closely associated with various neuropsychiatric disorders [2,20,22,26,29,30,137,138,139,140,141,142,143,144]. Strikingly, many genetic variants—including SNPs, single-nucleotide variants (SNVs), and de novo common and/or rare CNVs—in *SAPAP2* have been found to be associated with neurodevelopmental disorders such as ASD and ADHD [20,29,30,137,138,139,140,141,142,145], cognitive disorders such as developmental delay (DD)/ID and AD [22,142,143,145], and other disorders, such as schizophrenia [2,29,139,146,147,148] and OCD [26,144].

**Table 3:** Genetic variants in *SAPAP* genes associated with neuropsychiatric disorders.

Gene	Schizophrenia	ASD	OCD and Related Disorders	AD and Other Cognitive Disorders	ADHD and MDD
*SAPAP1*	Rare missense mutation c.1922A > G (a benign variant) [132]	Associated with the IGAP (International Genomics of Alzheimer’s Project) SNP rs8093731 in *Desmoglein-2/DSG2*; underexpressed in the entorhinal cortex, hippocampus, and frontal and temporal cortex of AD cases [28]	SNPs rs1116345 and rs34248 [144]	c.1397A > G, p. Asp466Gly, exon 8 in subcortical heterotopias [34]	SNPs rs2049161and rs16946051 associated with cognitive flexibility in ADHD [27] SNP rs12455524 in recurrent MDD [134]
	De novo CNV deletion [24]	Identified as an ASD-associated gene in a genome-wide network analysis [133]	Two SNPs located within an intron of SAPAP1 [25]	N/A	N/A
	N/A	N/A	Rare CNVs (62 kb duplication) [26]	N/A	N/A
*SAPAP2*	Damaging missense [147]	CNV 8:1626547:G:C [30]	SNPs rs6558484 and rs7014992 associated with OFC white matter volume [144]	8p23.3 deletion in DD/mental retardation [145]	N/A
	CNV duplication [146]	Rare de novo CNV duplications(704383-1521910) [29]	Rare CNVs (16 kb deletion) [26]	SNP rs34130287C within the first intron [149]	N/A
	SNV c.-69+9C.T, c.-69+13C.T, c.-69+47C.T, c.-69+55C.T [20]	SNP rs2906569 at intron 1, rs2301963 (P384Q) at exon 3 and several nonsynonymous variants [137]	N/A	SNP rs6992443 [150]	N/A
	N/A	Rare de novo CMV duplication 8:704383-1521910 [138]	N/A	SNP rs2957061 SNP chr8:1316870; minor allele frequency (a locus within *SAPAP2*) [22]	N/A
**Gene**	**Schizophrenia**	**ASD**	**OCD and Related Disorders**	**AD and Other Cognitive Disorders**	
*SAPAP2*	CNV deletion, 8p 23.2 and 8p 23.1 [139]	CNV deletion 8p 23.3 [151]	N/A	N/A	
	N/A	CNV deletion 8p 23.2 and 8p 23.1 [139]	N/A	N/A	
	CNV deletion, 8p23.3-p23.1 [148]	CNV duplication 8p23.3 (patients with de novo rearrangements) [140]	N/A	N/A	
	N/A	CNV duplication,8p23.3 (1, 499, 963–1, 854, 917) [141]	N/A	N/A	
*SAPAP3*	SNVs c.1141G > A; c.1759G > C; c.2309G > T; c.2578-11C > T [20]	CNV 1:35365700:G:A [30]	Increased frequency of rare nonsynonymous coding variants (in OCD and TTM) [152]	N/A	
	N/A	N/A	SNPs rs6662980–rs4652867 in grooming disorder [31]	N/A	
	N/A	N/A	SNP rs11264126 and two haplotypes containing rs11264126 and rs12141243 in TS [33]	N/A	
	N/A	N/A	SNPs rs11583978 and rs6682829 in early-onset OCD [32]	N/A	
*SAPAP4*	N/A	SNP located at the 20q11.21–q13.12 locus comprising the *Sapap4* gene [153]	N/A	De novo frameshift SNVs (c.2714_2715insCAGCTGG) insertion, N905Qfs, exon 12; c.2893T > G, p. Ser965Ala, exon 13 in subcortical heterotopias [34]	

Abbreviations: ASD, autism spectrum disorder; OCD, obsessive–compulsive disorder; AD, Alzheimer’s disease; TTM, trichotillomania; MDD, major depressive disorder; OFC, orbitofrontal cortex; TS, Tourette syndrome; DD, developmental delay; ADHD, attention deficit hyperactivity disorder; SNPs, single-nucleotide polymorphisms; SNVs, single-nucleotide variants; CNVs, copy number variants.

Although *SAPAP2* has been identified as a promising candidate gene of ASD, the potential role of *SAPAP2* in ASD is still unclear. A study using a murine model demonstrated that the deletion of *SAPAP2* leads to increased social interaction and aggressive behavior, along with impaired initial reverse learning and synaptic function [14], indicating that *SAPAP2* somehow modulates cognition. Correspondingly, a recent cross-species study not only revealed the role of *SAPAP2* in age-related working memory decline, but also established an association between *SAPAP2* and AD phenotypes at multiple levels of analysis [22], highlighting the possible importance of epigenetic dysregulation of *SAPAP2* expression in the pathophysiology of AD. As noted in the above studies, CDK5—one of the key kinases in AD—is involved in triggering amyloid-beta (Aβ)-induced ubiquitination and degradation of SAPAPs [70], while amyloid precursor protein (APP) is one of binding partners of Homer, which is indirectly linked to SAPAPs via SHANKs [7]. Gene overexpression can arise from de novo CNV duplication, and several autism risk genes in duplicated loci—such as *SAPAP2, SHANK3*, and *Neuroligins* 1 and 3—are all FMRP targets [29,117,121,138,140], suggesting a promising link between dysfunction of FMRP in regulating the expression of synaptic proteins and the development of ASD.

### 5.3. SAPAP3 and Neuropsychiatric Disorders

After the initial report of the features of *Sapap3*-mutant mice for OCD-like repetitive self-grooming behaviors [13], a higher frequency of rare nonsynonymous coding variants located in *SAPAP3* was found in OCD patients, providing the first clinical evidence and tentatively supporting the link between *SAPAP3* and OCD [152]. Several follow-up human genetic studies and other secondary analyses of GWAS have also noted that *SAPAP3* is a promising functional candidate gene for OCD and obsessive–compulsive spectrum disorders, such as trichotillomania (TTM) and Tourette syndrome (TS) [31,32,33,152], although some of the mutations identified in the above studies are more commonly found in association with grooming disorders and TTM than with OCD. Convergent evidence from functional imaging and neuropsychological studies has linked OCD symptoms to dysfunction of striatum-based circuitry, especially highlighting cortico-striato-thalamo-cortical circuitry [5,31,154]. Owing to their robust behavioral similarities and sophisticated neural circuitry shared with OCD and related disorders [13,82], *Sapap3*-mutant mice are now considered to be a well-established animal model for OCD and related disorders. Interestingly, a CNV within the *SAPAP3* gene was also identified in a study of about 200 individuals with ASD [30]—a spectrum of disorders that involve prominent compulsive-like repetitive behaviors overlapped with the behavioral phenotypes of OCD.

### 5.4. SAPAP4 and Neuropsychiatric Disorders

In addition to the aforementioned possible roles in the actions of psychostimulants, BD, and fragile X syndrome, *SAPAP4* is also associated with several other neuropsychiatric disorders, including ASD, early-onset cerebellar ataxia, and subcortical heterotopias [23,34,153]. An SNP located at the 20q11.21–q13.12 locus that encompasses the *SAPAP4* gene has also been linked with a potential role in the development of ASD [153]. *SAPAP4* is also implicated in early-onset cerebellar ataxia, according to an epigenetic study in which disruption of the *SAPAP4* gene by the chromosome translocation t (8;20) caused monoallelic DNA hypermethylation of the truncated *SAPAP4* promoter CpG island 820 bp downstream of the first untranslated exon of *SAPAP4* isoform a and perturbed expression of the *SAPAP4* gene [23]. A recent study has identified a de novo frameshift variation and a missense variant in *SAPAP4* from two families with different types of subcortical heterotopias—which are cortical malformations associated with ID and epilepsy [34]—pinpointing a novel role of *SAPAP4* in early cortical development. Although there is only an indirect link between *SAPAP4* and mood disorders such as BD [127], 22q13 duplications spanning *SHANK3* (a known interaction scaffold protein of SAPAP4) have been found in patients diagnosed with ADHD and BD, respectively [155], suggesting a possible role of *SAPAP4* in hyperkinetic disorders and mood disorders.

## 6. Mutational Studies in Murine SAPAP Models

Because increasing evidence has pointed to a link between SAPAP family members and some aspects of the pathophysiology of neuropsychiatric disorders, characterizing how the perturbed synaptic functions of SAPAPs are involved informs behavioral phenotypes related to neuropsychiatric disorders in mutant mice, revealing part of what makes neuropsychiatric disorders’ neurobiology.

To study the in vivo function of SAPAPs, mutant mice carrying *Sapap1–4* deletions have been generated (Table 4). *Sapap1*-mutant mice exhibit normal spontaneous locomotion and normal reverse learning ability, but subtle decreases in sociability and impaired association of PSD-95 with SHANK3 complexes [15,156], while *Sapap2*-mutant mice display enhanced social interaction ability, excessive aggressive behaviors, and abnormal synaptic morphology and transmission in the OFC [14]. In contrast, for reverse learning, two independent mutant lines show opposing phenotypes [14,156]. A recent study also found that deletion of SAPAP2 in mice leads to reduced alcohol consumption [21]. *Sapap1*- and -2-mutant mice all exhibit several forms of abnormal behavior relevant to schizophrenia, ASD, and cognitive disorders, such as deficits in learning, memory, and social behaviors.

Owing to the promising association between *SAPAP3* and OCD, much more attention has been given to *Sapap3*-mutant mice—a well-established OCD-relevant murine model displaying repetitive self-grooming behavior, augmented anxiety, cognitive inflexibility, imbalances between goal-directed and habitual behavior, selective deficits in behavioral response inhibition, insensitivity to reward devaluation, altered valence processing, hypolocomotion, disrupted sleep patterns, normal preference motivation for sucrose, and Pavlovian learning [13,102,104,106,157,158,159,160,161,162,163,164]. Convergent evidence from structural, biochemical, electrophysiological, and neural circuitry studies of *Sapap3*-mutant mice demonstrates and emphasizes the crucial role of SAPAP3 in corticostriatal synapses and striatum-based circuitry in OCD-like phenotypes. Surprisingly, neither cognitive inflexibility, augmented anxiety, nor aberrant habit formation are correlated with compulsive, repetitive behavior, implying that these different OCD-like behaviors in *Sapap3*-mutant mice probably involve complex or independent etiologies [157,159,160,162,163]. For example, the compromised PFC–striatal synaptic and circuitry function observed in *Sapap3*-mutant mice have been thought to be involved in their excessive compulsive and repetitive behavior [13,82,102].

Increased neural activity in prelimbic (PrL) and infralimbic (IL) regions of the medial PFC is associated with impaired reverse learning—usually representing cognitive inflexibility—in *Sapap3*-mutant mice [158]. Of particular interest, one study revealed an imbalanced cortical input to the central striatum in *Sapap3*-mutant mice, with more inputs from the secondary motor area (M2) and fewer inputs from the lateral OFC (LOFC) [103]. Notably, the role of SAPAP3 in the OFC appears to be highly heterogeneous.

**Table 4:** Characterization of *Sapap1–4*-mutant mice.

Target Protein	Murine Model Details and Background	Phenotype					
		Social Behaviors and PPI	Locomotion and Motor Ability	Compulsive and Repetitive Behaviors	Cognitive Function/ Learning and Memory	Anxiety, Depression, and Reward-Related Behaviors	Other Phenotypes
SAPAP1	*Dlgap1* KO (129/C57BL/6J)	↓ Sociability [15]	═ Locomotion [15]	↓ Grooming [15]	N/A	═ Depressive levels and sucrose preference [15]	↓ Digging behaviors [15]
	*Dlgap1* KO (129 S5/C57BL/6J)	N/A	═ Locomotion [156]	N/A	Slower reward collection latencies during reverse learning [156]	N/A	N/A
SAPAP2	*Dlgap2* KO exon 6 mutated (C57BL/6)	↑ Social approach and aggressive behaviors [14]	Novelty-induced hyperactivity [14]	NA	↓ Reverse learning [14]	N/A	N/A
	*Dlgap2* KO (129 S5/C57BL/6J)	N/A	═ Locomotion [156]	N/A	↑ Visual discrimination and reverse learning [156] Shorter reward collection latencies during reverse learning [156]	N/A	N/A
	*Dlgap2* KO exon 6 mutated (C57BL/6J)	N/A	N/A	N/A	N/A	↓ Alcohol consumption, preference, and binge-like drinking [21] ═ Sucrose preference [21]	N/A
**Target Protein**	**Murine Model** **Details and Background**	**Phenotype**					
		**Social Behaviors and PPI**	**Locomotion and Motor Ability**	**Compulsive and Repetitive Behaviors**	**Cognitive Function/Learning and Memory**	**Anxiety, Depression, and Reward-Related Behaviors**	**Other Phenotypes**
SAPAP3	*Sapap3* KO exon 3 mutated (C57BL/6J)	↓ PPI [109]	↓ Locomotion [104,105,157,159] ═ Locomotion [161] ↑ Locomotion by IC-DBS [165]	↑ Distinct forms of grooming [13,101,104,105,108,109,160,161,162,165,166] ↓ Adaptive grooming response [102] ═ Compulsive-like instrumental behavior [160] ↓ Grooming by IC-DBS [165]	↓ Reverse learning [106,157,158,162], fear learning, and fear extinction [164] ═ Pavlovian learning [157] and learning ability [106] ↑ Response lability [162] ↑ PFC activity associated with poor reverse learning [158]	↑ Anxiety behaviors [13,101,157] ═ Anxiety [160] Insensitive to the devaluation of a sucrose reward [159] ═ Sucrose preference and motivation [159] Altered reward processing and impaired habit learning [161] ↓ Acquisition of reward learning and goal-directed behaviors [164]	↓ Lactate and glutathione levels in the striatum [167] ↑ Serotonin turnover and metabolized dopamine ratios [108] ↓ D1/2/3 receptor density in the NAcc,↑DAT binding density in the striatum [109] ↓ SV2A [168] and mGluR5 availability [166] ↓ Response vigor [160]
	*Sapap3* KO, details not mentioned (C57BL/6J)	N/A	N/A	↑ Grooming [163]	↓ Reverse learning [163]	N/A	N/A
**Target Protein**	**Murine Model Details and Background**	**Phenotype**					
		**Social Behaviors and PPI**	**Locomotion and Motor Ability**	**Compulsive and Repetitive Behaviors**	**Cognitive Function/Learning and Memory**	**Anxiety, Depression, and Reward-Related Behaviors**	**Other Phenotypes**
SAPAP4	*Sapap4* KO replaced exons 3–6 with an ATG-YFP-STOP cassette (C57BL/6J)	═ Social interaction and recognition, ↓ PPI [17]	Hyperactivity, increased rearing behaviors ═ motor coordination and muscle endurance [17]	N/A	↓ Contextual and cued memory [17]	═ Anxiety [17] ↓ Time immobile in the forced swimming test and tail suspension test [17] Hypersensitivity to low doses of amphetamine [17] ↓ Sensitivity to cocaine and high doses of amphetamine [17,35]	Impulsive behaviors [17]
	*Dlgap4*^geo/geo^ exon trap vector integrated in intron 7 of the *Dlgap4* gene (C57BL/6J)	↓ Social interaction ↓ USV [16]	Hyperactivity, reduced rearing behaviors [16] ↑ Locomotion during the dark phase in home cages [16]	↓ Self-grooming [16]	↓ Working memory and spatial learning and memory [16]	↓ Anxiety [16]	═ General circadian activity. [16]
	*Dlgap4* KO exon 8 mutated (C57BL/6N)	N/A	N/A	N/A	N/A	N/A	Neuroanatomical defects in the dorsal telencephalon ↓ Cell counts across the entire brain Reduced in cortical layers II–IV and VI [34]
**Target protein**	**Murine Model Details and Background**		**Phenotype**				
			**Synaptic Morphology**	**Synaptic and Circuity Functions**		**mRNA or Protein Level**	
SAPAP1	*Dlgap1* KO (129/C57BL/6J)		N/A	N/A		Cortex (total proteins) ═ Homer1, PSD-95, NR2A/2B and SynGAP1 [15]	
SAPAP1	*Dlgap1* KO (129 S5/C57BL/6J)		N/A	N/A		N/A [156]	
SAPAP2	*Dlgap2* KO exon 6 mutated (C57BL/6)		OFC ↓ Spine density ↓ PSD length/thickness [14]	OFC ↓ mEPSC amplitude ↑ PPR [14]		Cortex (synaptosome proteins) ↓ NR1, GluR1,Homer1b/c, α and βCaMKⅡ [14] ═ NR2A/2B, GluR2, SHANK3 and PSD95 [14]	
	*Dlgap2* KO (129 S5/C57BL/6J)		N/A	N/A		N/A [156]	
	*Dlgap2* KO exon 6 mutated (C57BL/6J)		N/A	N/A		N/A [21]	
**Target Protein**	**Murine Model Details and Background**	**Phenotype**					
		**Synaptic** **Morphology**	**Synaptic and Circuity Functions**			**mRNA or Protein Level**	
SAPAP3	*Sapap3* KO exon 3 mutated (C57BL/6J)	Striatum ═ Spine density and PSD length,↓PSD thickness [13] Associative striatum ↓ Axon caliber ═ Density of myelinated axons [96]	Striatum ↓ fEPSP amplitude, ↑ fEPSP (NMDA), ═ PPR [13] ↓ PS amplitude and PPR, ↓ eEPSC amplitude and ↑ eEPSC PPR (D2 MSNs); ↑ synaptic depression [99] ↓ AMPA/NMDA, ↓ mEPSC amplitude/ frequency (AMPA), ═PPR, ↑ silent synapses [95] ↓ N2 field potential amplitude, ↑ PPR [169] ↑ Baseline firing rate [102] ═ mEPSC (striatopallidal MSNs), ↓ qEPSC frequency; ═ qEPSC frequency/amplitude (thalamostriatal synapses), ═ AMPA/NMDA, ═ PPR [82] ↓ Firing rate and spike probability (MTEP treatment), ↑ spike probability, ↑ event amplitude, ↑ mEPSC frequency (MTEP treatment) [101] Striatum: ═ SPN and FSI I–O curves, EPSC amplitude: ↑ excitatory drive to FSIs, ↓ LOFC inputs to SPNs, ↑ M2 input to central striatum [103] Inhibition of striatal cells increases grooming behavior; inhibition of striatal indirect pathway neurons decreases grooming [105]		Striatum ═ mEPSC amplitude/frequency (PV+ cells), ═ PPR, ↓ AMPA/NMDA (D1-, D2-MSNs), ↓ rectification index (D2-MSNs), ↓ cumulative probability (M1/M2-to-DLS AMPAR currents) [104] LOFC ↓ LFP oscillation power, ↑ firing rate (putative interneurons), ↑ bursting activity (putative pyramidal neurons) [170] Altered OFC-striatal circuit [102,106] and corticostriatal circuitry [169] ↑ Activity in LOFC excitatory neurons/activity in LOFC GABAergic interneurons in the early-reversal stage [106]	Striatum (PSD proteins) ↑ NR1/2B,↓ NR2A, ═ PSD-95/93 and SHANK [13] SAPAP4 is present at thalamostriatal synapses but not corticostriatal synapses [82] ↓ mGluR5/Homer interaction [101] mRNA *Sapap3* is the sole *Sapap* that is highly expressed in the striatum, *Sapap4* is the next most abundant [82]	
**Target Protein**	**Murine model Details and Background**	**Phenotype**					
		**Synaptic Morphology**		**Synaptic and Circuity Functions**		**mRNA or Protein Level**	
SAPAP3	*Sapap3* KO details not mentioned (C57BL/6J)	N/A		Altered dmPFC-DMS circuit in compulsive groomers [107]	Distinct patterns of abnormal LOFC activity during grooming and reverse learning [163]	N/A	
SAPAP4	*Sapap4* KO replace exons 3–6 with an ATG-YFP-STOP cassette (C57BL/6J)	mPFC ↓ Spine density ═ PSD length/thickness [17] NAc (shell) ↓ Spine density ↓ Numbers of synapses with AMPARs [35]		NAc (shell) ↓ Field population spike amplitude, ↓ mEPSC frequency/amplitude, ═ PPR, NMDA/AMPA [35]	N/A	PFC PSD proteins: ↑ SHANK/SHANK3, ↓ PSD-95 and GluR1/2, ═ PSD93, Homer 1b/c, NR1, NR2A/2B, mGluR5 and SHANK1/2 [17] **mRNA:***Sapap4* re-expression rescues *Shank3* mRNA expression in cultured PFC neurons of KO mice [17]	
	*Dlgap4*^geo/geo^ exon trap vector integrated in intron 7 of the *Dlgap4* gene (C57BL/6J)	Hippocampus (CA1) ↓ Apical dendritic path length, intersections and branching points, ═ Basal dendrites, ↓ stubby spines, ↑ PSD size [16]		Hippocampus (CA1) ═ fEPSP, ↑ mEPSC amplitude, ═ mEPSC frequency, ↓ NR2A-EPSC, ↓ LTD(LFS), ═ LTP Hippocampus and PFC ↑ Oscillatory events’ amplitude, ↓ oscillatory events’ duration [16]	N/A	Hippocampus (PSD proteins) ═ NR1, NR2A-C, GRIA1-4, mGluR5, Actn2, Akap1, Baiap2, Dlg1-4, GKAP, Grip1, Magi1, Nsmf, SAPAP1-3, pan-SHANK, SHANK3, aCaMKII, SynGAP1 [16]	
	*Dlgap4* KO exon 8 mutated(C57BL/6N)	N/A		N/A	N/A	*Sapap4* mRNA expression throughout the murine cortical wall at E14.5 [34]	

Abbreviations: ↓, reduced; ═, unaltered; ↑, increased; N/A, not available; M1, primary motor cortex; M2, secondary motor cortex; DLS, dorsolateral striatum; DMS, dorsomedial striatum; LFS, low-frequency stimulation; PFC, prefrontal cortex; dmPFC, dorsal lateral prefrontal cortex; dmPFC, dorsal medial prefrontal cortex; LOFC, lateral part of the orbitofrontal cortex; LOFC, lateral orbitofrontal cortex; OFC, orbitofrontal cortex; IC, internal capsule; NAc, nucleus accumbens; NAcc, nucleus accumbens core; PV, parvalbumin; MSNs, medium spiny neurons; SPN, spiny projection neuron; USVs, ultrasonic vocalizations; PPI, prepulse inhibition; MTEP, mGluR5 antagonism; KO, knockout; KD, knockdown; PSD, postsynaptic density; SV2A, synaptic vesicle protein 2A; PPR, paired-pulse ratio; mEPSC, miniature excitatory postsynaptic current; LTD, long-term depression; LTP, long-term potentiation; fEPSP, field excitatory postsynaptic potential; eEPSC, evoked excitatory postsynaptic current; qEPSC, quantal excitatory postsynaptic current; PS, population spike; EPSC, excitatory postsynaptic current; N2, second negative peak; I–O curves, input–output curves; LFP, local field potential; FSI, fast-spiking interneuron; E14.5, embryonic day 14.5; D1/2/3, dopamine receptor 1/2/3; DAT, dopamine transporter.

The LOFC is involved in both repetitive behaviors and reverse-learning deficits, but with disparate patterns of abnormal activity. Optogenetic stimulation of the LOFC’s pyramidal neurons or LOFC–striatum circuitry reduces compulsive grooming in *Sapap3*-mutant mice [102]. However, inhibition of LOFC GABAergic interneurons results in increased activity in the LOFC’s pyramidal neurons and constitutes a direct pathway that leads to the impairment of reverse learning in *Sapap3*-mutant mice [106].

Three independent *Sapap4*-mutant mouse lines generated using different deletion strategies have been used to investigate the in vivo functional roles of SAPAP4 [16,17,34,35]. Of these, one mouse line has not been tested for all behaviors [34]; the other two show both shared and distinct defects at the synaptic and behavioral levels [16,17,35]. These two lines both show robust hyperactivity and memory deficits [16,17]. Whereas *Dlgap4*^geo/geo^ mice with an exon trap vector integrated in intron 7 of the *Sapap4* gene show synaptic defects in the hippocampus and display impaired social interaction, diminished anxiety, and normal grooming behavior [16]—in which the social defects phenotype resembles the ASD-like behaviors observed in various *Shank3*-mutant mice [57]—mutant mice carrying *Sapap4* deletions of exons 3-6 have profound synaptic defects in the NAc and exhibit robust hyperactivity, reduced depression-like behavior, altered sensitivity to amphetamine and cocaine, and reduced sensory responses, without deficits in anxiety and social interaction [17,35], and the behavioral phenotype of this line replicates the BD manic-like hyperkinetic behaviors seen in *Shank3* transgenic mice [155]. Consistently, SHANK3 expression in the PSD is significantly upregulated in mice carrying *Sapap4* deletions of exons 3-6 [17]. In addition, a recent study has shown that heterozygous mice with *Sapap4* deletions (i.e., removal of exon 8) exhibit decreased size in several brain structures, with the hippocampus, corpus callosum, anterior commissure, thalamus, and cortex affected [34], suggesting the potential role of SAPAP4 during brain development. Given that the disruption of SAPAP4 in *Dlgap4*^geo/geo^ mice mainly affects the C-terminal region of SAPAP4 (including the binding site for SHANKs) and results in almost total loss of interaction with SHANKs, and that the ablation of exons 3–6 leads to the loss of the vast majority of the SAPAP4 protein (especially the N-terminal region), these two studies provide insights into how different domains or isoforms of the *Sapap4* gene may elicit domain- or isoform-specific synaptic and behavioral defects and be involved in different neuropsychiatric disorders.

## 7. Conclusions

Emerging evidence indicates that SAPAPs are multifaceted postsynaptic scaffold proteins at excitatory synapses; they are believed to control excitatory synaptic formation and maturation, and play crucial roles in homeostatic plasticity by regulating the accumulation and turnover of glutamatergic receptors and scaffold proteins at synaptic sites [4,86], which may involve FMRP, ubiquitination, and actin cytoskeleton dynamics (Figure 2). Although evidence from direct association studies between neuropsychiatric disorders and SAPAPs is limited, there is ample convergent evidence linking dysfunction of SAPAPs to various cognitive deficits and strongly supportive of the important roles of SAPAPs in neuropsychiatric disorders. Findings from human genetic studies and mutant murine models are coalescing into a picture of the molecular networks that, when dysregulated or disrupted, may lead to synaptic dysfunction and, ultimately, be responsible for various neuropsychiatric disorders, such as OCD, ASD, ADHD, BD, schizophrenia, addiction, AD, and other cognitive disorders.

Undoubtedly, *Sapap*-mutant mice could be useful tools with which to dissect the effects of SAPAP disturbances on neuronal and circuitry function, and to decipher the neurobiological mechanisms underlying behavioral abnormalities seen in neuropsychiatric disorders, including genetically overlapped psychiatric disorders. Given that different SAPAP members have shared and distinct expression patterns in different developmental stages, brain regions, cell types, and circuitry, with differential regulation of gene expression at the transcript, translational, and epigenetic levels, as well as different interacting partners and signaling molecules and possible compensatory expression of other SAPAP members, it will be very important to clarify the physiological functions of the individual SAPAPs, along with how disrupted SAPAPs may result in molecular, synaptic, and circuitry defects and related abnormal behaviors with regard to neuropsychiatric disorders.

Therefore, future studies on *Sapap*-mutant mice should take into consideration the alternative splicing variants of the gene, the expression timing, the site, and the cell type where the gene mutation exerts its primary effects, eventually compromising the specific neural circuit. Importantly, the significance of the specific neural circuits to individual SAPAP proteins and behavioral abnormalities relevant to neuropsychiatric disorders still needs to be further explored by combining the manipulation of specific circuitry with conventional and conditional gene manipulation strategies.

## Figures and Tables

**Table 1:** Changes of SAPAPs’ mRNA and/or protein expression associated with neuropsychiatric disorders.

Gene	Regulation	Expression	Brain Regions	Animal Models/Patients	References
*SAPAP1*	Upregulation	Protein	Cortex or hippocampus	*Fmr1*-KO mice	[117]
	Upregulation	mRNA or protein	Rats: NAc and hippocampus; patients: NAc	Schizophrenia rat model and patients	[115]
SAPAP2	Upregulation	Protein	Cortex or hippocampus	*Fmr1*-KO mice	[117]
SAPAP3	Upregulation	Protein	Cortex or hippocampus	*Fmr1*-KO mice	[117]
	Upregulation	mRNAor Protein	NAc	Syrian hamsters after aggressive experience	[119]
	Downregulation	Protein	OFC and mPFC	*Fmr1*-KO mice	[118]
	Upregulation	Protein	Mice: cortex and hippocampus; patients: cortex	Epilepsy patients and murine models	[116]
*SAPAP4*	Upregulation in the hippocampus	mRNA	Hippocampus	*Fmr1*-KO mice	[123]
	Downregulation	mRNA	Amygdala	Taste-aversion-resistant rats after cocaine exposure	[126]

**Table 2:** Epigenetic dysregulation of the expression of *SAPAP* genes associated with neuropsychiatric disorders.

Gene	Regulation	Expression	Brain Regions/Cells	Animal Models/Patients	References
*SAPAP2*	Upregulation (with lower DNA methylation)	mRNA	Hippocampus	PTSD rat model	[112]
	Upregulation (with lower DNA methylation)	mRNA	dlPFC or NAc	Patients with alcohol dependence	[21]
	Downregulation (related to DNA methylation)	mRNA protein	dlPFC	AD patients	[22]
*SAPAP3*	Downregulation (by absence of histone deacetylases)	mRNA	Frontal cortex and striatum	Mice deficient in histone deacetylases 1 and 2	[128]
*SAPAP4*	Disruption of *SAPAP4* by the chromosome translocation results in monoallelic hypermethylation of the truncated *SAPAP4* promoter CpG island	N/A	Cerebellum	Early-onset cerebellar ataxia patients	[23]
	Identified as a target of the BD-associated microRNA miR-1908-5p	N/A	Neural progenitor cells	BD patients	[127]

Abbreviations: AD, Alzheimer’s disease; BD, bipolar disorder; PTSD, post-traumatic stress disorder.
